# The Impact of Diet and Physical Activity on Bone Health in Children and Adolescents

**DOI:** 10.3389/fendo.2021.704647

**Published:** 2021-09-13

**Authors:** Patrizia Proia, Alessandra Amato, Patrik Drid, Darinka Korovljev, Sonya Vasto, Sara Baldassano

**Affiliations:** ^1^Department of Psychological, Pedagogical and Educational Sciences, Sport and Exercise Sciences Research Unit, University of Palermo, Palermo, Italy; ^2^Faculty of Sport and Physical Education, University of Novi Sad, Novi Sad, Serbia; ^3^Department of Biological, Chemical and Pharmaceutical Sciences and Technologies (STEBICEF), University of Palermo, Palermo, Italy

**Keywords:** macronutrients, exercise, bone mass, gut peptides, hormones, children, adolescent

## Abstract

There is growing recognition of the role of diet and physical activity in modulating bone mineral density, bone mineral content, and remodeling, which in turn can impact bone health later in life. Adequate nutrient composition could influence bone health and help to maximize peak bone mass. Therefore, children’s nutrition may have lifelong consequences. Also, physical activity, adequate in volume or intensity, may have positive consequences on bone mineral content and density and may preserve bone loss in adulthood. Most of the literature that exists for children, about diet and physical activity on bone health, has been translated from studies conducted in adults. Thus, there are still many unanswered questions about what type of diet and physical activity may positively influence skeletal development. This review focuses on bone requirements in terms of nutrients and physical activity in childhood and adolescence to promote bone health. It explores the contemporary scientific literature that analyzes the impact of diet together with the typology and timing of physical activity that could be more appropriate depending on whether they are children and adolescents to assure an optimal skeleton formation. A description of the role of parathyroid hormone (PTH) and gut hormones (gastric inhibitory peptide (GIP), glucagon-like peptide (GLP)-1, and GLP-2) as potential candidates in this interaction to promote bone health is also presented.

## Introduction

Bone tissue is a real organ in constant change, with both locomotive and supportive functions. It can be stiff but also flexible. These characteristics depend on the bone’s material composition. The bone grows in length with endochondral ossification processes and in size with the modeling process of periosteal and endosteal surfaces (1). In particular, formation and resorption allow increasing bone mass and changing tissue density. The cartilaginous epiphyseal plate is the space where the bone grows in length as a result of chondrocyte proliferation and maturation, replacing the cartilage tissue with bone construction. In order to allow the complete ossification process, osteoclasts and osteoblasts are directed, by blood vessels, into the new cartilage tissue (2). This process runs constantly till the early twenties until the growth plate cartilage is replaced completely by new bone (3). When the bone stops to grow in length, it keeps increasing in cross section in order to adapt to different mechanical loads and to compensate for bone loss. Osteoclasts and osteoblasts perform different tasks during the process of bone construction; the former are responsible of shaping the long bone’s outer surface by building on circumferential lamellae. Osteoclasts are responsible for endosteal bone surface resorption and overall bone remodeling. The process of bone shaping and remodeling is sex and age dependent (4, 5), leading to the bone with an optimal size, shape, and architecture to withstand the normal physiological loads imposed on it. Failure to gain a sufficiently strong skeleton during growth may predispose to bone fragility later in life.

Thus, what are the key factors that may positively influence skeletal health in children and adolescents to maximize peak bone mass? Lifestyle, including diet and adequate physical activity, is an external factor of bone mass development that during growth and also during adulthood can help in the building of a strong adult skeleton (6, 7). Most of the literature that exists for children and adolescents about the effects of diet and physical activity on bone health is translated from studies conducted in adults. Thus, so far, relatively few studies have investigated the association between macronutrients or physical activity and bone mass depending on whether they are children or adolescents. Therefore, there are still many unanswered questions about what type of diet and physical activity may positively influence skeletal development. This review focuses on bone requirements in terms of nutrients and physical activity during childhood and adolescence. It explores the contemporary scientific literature that analyzes the impact of diet together with the typology and timing of physical activity that could be more appropriate depending on whether they are children and adolescents in order to assure an optimal skeleton formation. A description of the role of parathyroid hormone (PTH) and gut hormones (gastric inhibitory peptide (GIP), glucagon-like peptide (GLP)-1, and GLP-2) as potential candidates in this interaction to promote bone health is also presented.

## The Influence of Macronutrients and Micronutrients on Bone Health

Nutrition is an essential process for healthy growth and development of the skeleton. Diets are mainly composed of macronutrients (protein, fat, and carbohydrates) and also of micronutrients like dietary calcium, phosphorus, and vitamin D. Together, they are essential factors in promoting bone health and preventing bone loss. In the following paragraph, we will focus on studies that have investigated the influences of macronutrients and micronutrients on the bone to assure an optimal skeleton formation.

### Proteins

Protein consumption exerts a beneficial effect on bone health in adults, while very little is known on short- or long-term effects of protein supplementation on bone turnover and bone development in children and adolescents. As far as we know, the recommended dietary allowance (RDA) in healthy children and adolescents is strictly derived from studies conducted in adults (8, 9) in which the recommended protein intake varies from 1.03 g/kg for all individuals to 0.97 g/kg per day for children aged 18–24 months.

In 2012, the EFSA Panel gave a scientific opinion on setting the dietary reference value (DRVs) for protein, which also takes into account protein quality. The daily amount of protein is calculated according to age and sex and set the population reference intake (PRI) that for adults goes from 0.66 to 0.8 g/kg body weight per day. In older adults, the PRI goes from 0.8 to 1.0 g/kg body weight per day in relation to low energy requirement of sedentary elderly or more energy dense protein for physically active groups. In infants and children, the EFSA Panel suggested a PRI from 1.65 to 1.1 g/kg body weight per day for the first year of life; between 1.15 and 0.9 g/kg body weight per day from 2- to 5-year-old children; and from 5 to 18 years old, the PRI goes from 1.1 to 0.8 g/kg body weight per day (10). Moreover, not much data are present for children above or under average weight and young athletes; and this might cause insufficient coverage in protein and energy requirements along children’s growth (11) in order to assure an optimal skeleton formation (12).

Usually, in adult athlete population, protein RDA is defined by 1.2 and 1.7 g/kg/day (13), but a recent study affirms that protein intake in young boys before pubertal maturation should increase up to 2 g/kg/day in order to obtain higher bone acquisition as positive effect of physical activity because the positive effect on skeleton health is due to protein intake instead of calcium intake (12, 14). Thus, more research to clarify the point is necessary. Another open question is whether recommended protein intakes should be increased on the basis of the source of protein. Many conflicting studies have tried to examine the beneficial or detrimental role of protein intake on bone health, based on the source of protein (animal *vs.* vegetable) and the amount of protein ingested (high *vs.* low quantities) (15), namely, the sulfur content that may vary in sulfur-containing amino acids present in the protein source (animal or vegetable proteins). Therefore, a greater production of sulfuric acid could induce low- or mild-grade metabolic acidosis that, in turn, may have a negative effect on bone remodeling by enhancing bone resorption (16). However, when a balanced diet with adequate intake of calcium, vitamin D, fruits, vegetables, and protein of animal source is ingested, this does not exert a detrimental effect on the bone and improves bone health (17) On the contrary, in pubertal girls, when calcium intakes were less than 675 mg/day, the high-protein intake, especially from animal sources, had a negative effect on bone mass accrual deposition (18). It is quite clear, from literature, that sulfur amino acids play critical roles in metabolism and overall health maintenance. Animal-derived foods are a good source of sulfur-containing amino acids (19). Besides their role in protein synthesis, methionine and cysteine are precursors of important molecules (20). The most popular food for children and adolescents that are a good source of dietary sulfur-containing amino acids are chicken and beef, but also white eggs mainly present in products like omelet, frittata, crepes, mayonnaise, biscuits, and cakes. So the positive effects of protein on the bone are exerted only if the energy requirements are satisfied by the carbohydrates and fats; otherwise, proteins are catabolized to sustain the energy demands. Thus, adequate intake of fat and carbohydrates to maintain bone health is essential (21, 22).

The protein needs of adult athletes are higher than those of non-athletes (23, 24). Thus, we do not know the quantity of acid formed, as sulfate may have a negative effect on the young skeleton. Thus, further studies about the impact of the source of protein origin (animal or vegetable) on the bone of child athletes or highly active children would be of interest. Moreover, this concept is closely linked to food habits. In fact, there is some evidence that suggests substantial differences in the consumption of food containing vegetable or animal proteins on the basis of age. Children ranging in age from 2 to 13 years (in both sexes) are used to consuming more red meat, poultry, or fish daily. Adolescents, from 14 to 16 years old, especially girls, have a marked decrease in animal protein consumption. However, the average intake of the meat was generally higher for males (100 g/day) than for females (80 g/day) (25).

Furthermore, it is important to underline that proteins may affect the bones at various levels: 1) they represent the major component of the bone matrix, and 2) they impact calcium excretion and absorption (26) and serum concentrations of insulin-like growth factor (IGF-1) (25). Therefore, adequate protein intake may be crucial for young athletes, for example, those involved in non-weight-bearing activities like swimming, who seem to be at increased risk for suboptimal peak bone mass development (27). Western diet often accounts for being responsible for osteoporosis or bone fracture due to the high protein content associated with hypercalciuria (21). Thus, as protein intake increases, there is an increase in urinary calcium excretion, with most subjects developing negative calcium balance as well as increased risk of fracture (28). Therefore, it is essential that the intake of protein should be adequate in order to fully realize the benefit of each nutrient on the bone. In fact, for example, proteins are able to modulate the IGF-1 levels that, in turn, would impact on both the skeletal muscle and bone, reducing fracture risk and increasing speed of recovery following a bone injury (29). In addition, there is evidence of a positive bone turnover response after protein intake shortly after intense exercise in adolescents. A study reveals a significant reduction in marker of bone resorption (CTX) after protein intake based on whey protein beverage following intense physical activity such as swimming. Thus, protein intake, after exercise, is important for the increase of bone mass during childhood, in particular if the subjects are active, or athletes (30). On the other hand, if protein intake is low (0.7–0.8 g/kg), the amount of PTH levels will increase in blood, while if moderate (1.0–1.5 g/kg), it is associated with normal calcium metabolism, without altering bone homeostasis. In gymnasts bone resorption was reduced by a high carbohydrate meal consumed 90 minutes before the training but not by a high protein meal (6). After all, any lifestyle stratsegy that can promote bone buildup in children, without affecting whole body homeostasis, is beneficial because it will drive towards higher peak bone mass and improvement in bone mineral density (BMD). Future research should focus on the long-term benefits of protein intake on blood markers of bone turnover in association with exercise especially in children and adolescents practicing low- and high-impact physical activity.

### Fat and Carbohydrates

Many studies have explored the effects of fat and carbohydrates intake on bone health in children by focusing on calcium absorption rather than its direct effect on BMD, bone mineral content (BMC), and bone remodeling. Thus, we will first focus on it to briefly make the point on what is known and the importance with respect to the impact for bone growth in children. The studies have been carried out to understand whether or not fat or carbohydrate hindered calcium absorption in the intestine. Until now, calcium absorption has been most studied with respect to its impact on bone metabolism. As regards calcium absorption and fat, most of the studies were carried out on animal models fed with diets that contained a variable percentage of saturated fats (SFs) (5%, 14%, 28%, and 45%). The results have shown poor calcium absorption probably due to the formation of non-digestible calcium and saturated fatty acids (SFA) complexes in the intestine. In particular, a reduction in calcium absorption started when the diet administered contained up to 28% of fat, while there was a dramatic decrease in intestinal absorption of calcium when it reached 45% of fat. As a consequence, high-fat diet consumption in animals and humans is associated with reduction of BMD and bone strength (31). Actually, adverse microstructure changes occur in the cancellous bone compartment. Corwin and collaborators based on data from the Third National Health and Nutrition Examination Survey (NHANES III) conducted a study on 14,850 subjects, confirming a negative association between SF intake and BMD in both men and women (32).

Regarding the impact of carbohydrate intake on calcium absorption, numerous studies investigated the role of monosaccharide (particular glucose) and disaccharides (particular sucrose), showing an effect on the renal metabolism at the level of the distal tubule region of the nephron, therefore influencing the reabsorption of calcium (28, 33–35). In particular, there will be a large increase in renal excretion following glucose ingestion. For example, Ericsson et al. in human studies pointed out an exaggerated loss of calcium through the urine following the intake of a solution of glucose. The lactic acid formed by the osteoclast following the increase in glucose intake could induce the dissolution of calcium and magnesium from the bone surfaces, with consequent increase of urine calcium excretion with respect to the normal range. Other studies on both human and animal models showed that the insulin spike triggered by the ingestion of a high amount of glucose was directly proportional to the urinary levels of calcium excretion (36). These data suggest that more attention should be paid to the children’s diet. The question is why reduction of calcium absorption induced by fat and sugar could affect the growing of a healthy bone in children. Because as they grow, there is a decrease in the consumption of milk and a concomitant increase in the consumption of unhealthy food rich in SF and soft drinks, often attributed to gaining independence in choosing what to drink. In particular, data from the Continuing Survey of Food Intakes report (CSFII 1994–1996) suggest that with aging, there is a reduction in milk intake with a ratio of about 30 ml with a concomitant increase of approximately 126 ml in sweetened drinks. This is associated with an increase in caloric intake, of approximately 30 kcal, and a concomitant reduction in calcium consumption of 34 mg for each 30 ml of milk displaced (16). In young children, both athletes or not, the increased intake of soft drinks or the consumption of food rich in SF could have a negative impact on bone health and performance due to the impact of these on calcium absorption. This is the reason why it may be important to suggest using unsweetened drinks (water and milk) or orange juice, or sports drinks but only with small amounts of carbohydrate (<2%) and moderate amounts of sodium (37) and fortified with calcium in order to obtain rehydration and reduce or prevent loss of BMD. Furthermore, following a healthy diet rich in unsaturated fat is also suggested. For example, animal studies have shown that polyunsaturated fatty acids (PUFAs) such as omega-3s have been shown to reduce bone resorption and increase bone formation (38). The positive effect of a diet high in PUFA was confirmed in humans. The subjects involved in the study were assigned to three different groups of intakes consisting of 8%–13% of SFA, 12%–13% of monounsaturated fatty acids (MUFA), and 9%–17% of PUFA for a period of 6 weeks. There was no change in levels of bone-specific alkaline phosphatase (BSAP), selected as a marker of bone formation, across the three diets, while there was a reduction of bone resorption following the PUFA-enriched diet (39). The results indicated that dietary PUFA may have a protective effect on bone metabolism. Studies are necessary to see the impact of unsaturated fat on bone remodeling in children. Carbohydrates are present in fruits and vegetables and influence the absorption of calcium and therefore might influence bone growth. For example, chicory and artichokes have a high content of non-digestible carbohydrates (they are not digested by mammalian enzymes) called fructans such as inulin, which increases the absorption of calcium (40, 41). In fact, a study conducted on 9- to 13-year-old boys and girls, for a total of 12 months, with 8 g/day supplementation of inulin-type fructan, showed that calcium absorption, BMC, and BMD were significantly higher in the inulin-type fructan-supplemented group than in the placebo (supplemented with maltodextrin) control group (42). As far as the mechanism of action is concerned, it was suggested that these types of molecules, which are not digested, will reach the colon where they will be fermented, producing organic acids capable of reducing the pH by increasing the solubility and availability of calcium (43). The daily intake of carbohydrates should not go down 50%–55% of the diet. Adam-Perrot et al. confirmed that consumption of a low-CHO diet leads to an increase in urinary calcium loss and a decrease in markers for bone formation. Moreover, in adults, low-CHO diets lead to an increased consumption of animal protein, generating an acidosis that promotes calcium mobilization from the bone, finally leading to an increase of urinary calcium (44). The lack of a consistent definition for “low carbohydrate diets” complicates efforts to compare the results of the studies already published in this field. While it is known in the adult population that when we refer to “very low carbohydrate diets,” we are talking about less than 70 g/day based on the proportion of energy intake; a diet containing 200 g of carbohydrate might be classified as moderately low for a 2.000 calorie intake, moderate carbohydrate for 1.500 calories, and high carbohydrate for 1.200 calories (45).

In conclusion, there is a need for studies about the effects of carbohydrates and fat on bone metabolism in children to see their impact on bone growth. Future studies should focus on both the short- and long-term benefits of carbohydrate and unsaturated fat consumption/supplementation on bone remodeling and BMD in children and adolescents, by looking also to those children participating in intensive training.

### Micronutrients

Vitamin D and calcium are known to play key roles in bone health. Optimal calcium intake is estimated to be 400 mg/day from birth to 6 months, 600 mg/day in infants (6 to 12 months), 800 mg/day in young children (1–5 years) and 800–1,200 mg/day for older children (6–10 years), and 1,200–1,500 mg/day for adolescents and young adults (11–24 years) (46, 47). Milk and milk products such as 125 g of yogurt or 50 g of cheese allow the intake of about 300 mg of calcium. However, alternative calcium sources are orange juice, some vegetables such as cabbage family or large leafy vegetables, spinach, legumes, and some cereals, which contribute to the daily calcium intake for about 10%. However, vegetables having high calcium content have reduced calcium bioavailability due to the high concentration of oxalates (48). In general, reducing the intake of dairy products below the recommended daily doses can have a negative impact on the bone not only due to the lack of calcium but also due to other micronutrients such as phosphorus, potassium magnesium, or vitamins (B2 and B12, A, and D) (49).

The effects of calcium alone and together with vitamin D supplementation on the bone health of children have been investigated by different trials using also twin children. Johnston and collaborators showed that in prepubertal twins supplemented with 1,612 mg of calcium daily for 3 years, there was an increase of BMD with respect to the twin used as a control and supplemented with 908 mg of calcium in spite of the same intake of all nutrients and the equivalent level of physical activity (50). In a double-blind randomized control trial, it was investigated whether there was a differential response to calcium supplementation in elite prepubertal gymnasts and schoolchild controls. It was found that 1,250 mg daily of calcium supplementation, with a low level of physical activity, had only a small positive change in tibia trabecular volumetric BMD in the control group. Moreover, it was found that there was no beneficial effect of additional calcium in prepubertal gymnasts who already consume their recommended nutrient intake of calcium (51). In a randomized trial, adolescent girls, aged 12 years, were enrolled and supplemented with daily calcium, 800 mg of calcium carbonate and 400 IU vitamin D for 12 months. Daily calcium and vitamin D supplementation promoted greater trabecular BMC and volumetric BMD acquisition in these preadolescent girls (52). Accordingly, Greene and collaborators confirmed that 800 mg of calcium and 400 UI of vitamin D supplementation, every day for 6 months, increased trabecular density and strength strain index and increased the tibial cortical area in female identical twins, aged 9 to 13 years (53). However, a meta-analysis study showed that increased dietary calcium/dairy products, with and without vitamin D, significantly increased total body and lumbar spine BMC in children with low baseline calcium intakes (54). So it seems that calcium and vitamin D supplementation above the recommended nutrient intake has a modest influence on the bone especially in active prepubertal children. On the other hand, calcium supplementation seems to be beneficial for the child population with low daily intake.

Children aged between 3 and 17 years seem to take more than 50% of the recommended daily dose of calcium through dairy products as confirmed by studies carried out in France (2005–2007) and the United States (49). The latter pointed out that children aged between 2 and 18 years take about 950 mg/day through dairy products (especially milk and cheese), which represents the main source (55). The optimal quantity of phosphorus to take is in a ratio of 2:1 to calcium (in favor of the latter) since it could have the opposite effect. Sodium may also have an effect on urinary calcium excretion, as both sodium and calcium compete for reabsorption in the renal tubules. It is estimated that for every 2,300 mg of sodium excreted, approximately 50 mg of calcium is lost in women (56). The influence of short-term calcium supplementation in adult athletes during exercise on bone remodeling has been investigated. Short-term supplementation with calcium, 60 min before physical activity, did not appear to affect bone resorption (57), but this could be due to the quantity of supplementation or the time of consumption before the trial. In fact, a calcium supplement of 1,000 mg, taken 30 min before exercise, did not affect bone resorption in competitive adult male cyclists, while 1,350 mg of calcium taken 90 min before the exercise reduced bone resorption in competitive adult female cyclists (58). As regards vitamin D, the general recommended daily dose is 200 IU/day for children. Specifically, the RDA for children up to 1 year is 400 IU/day, which increases up to 600 IU for children aged 1 year or older. Also in this case, the upper limits are set beyond which it could have a detrimental effect, specifically for children up to 6 months with daily dose of 1,000 IU, from 6 to 12 months of age with daily dose of 1,500 IU, from 1 to 3 years of age with daily dose of 2,500 IU, from 4 to 8 years of age with daily dose of 3,000 IU, and from 9 and 18 years with daily dose of 4,000 IU (48). However, it should be kept in mind that in addition to calcium intake, there are also other factors that affect bone health such as hereditary and environmental factors.

## Dietary Composition

Bone health could be more influenced by dietary long- or short-term changes rather than specific nutrients, although there are insufficient studies to prove it.

The HELENA study failed to show, in Spanish adolescents, the association between Mediterranean diet and BMC (59) as well as a study of Monjardino et al. (60), which found no association between forearm BMD and different dietary patterns. The work of Shin et al. observed that adolescents in the highest tertile with a dietary pattern rich in cereal and milk score significantly a reduced chance of having low BMD than do those in the lowest tertile (61). The association among dietary patterns, physical activity BMC, and BMD needs to be further explored especially in children. In relation to this issue, Muñoz-Hernandez et al. showed that moderate-to-vigorous physical activity and less time for sedentary behavior seem to improve bone health in overweight or obese children with related poor adherence to the Mediterranean dietary pattern (62).

In the review of Mariotti and collaborators, they indicated that classic vegetarian diets provide more than adequate protein and amino acids with respect to the DRVs; furthermore, children who are consuming sufficient energy to cover their necessities for growth should automatically reach sufficient protein intake and protein variety from vegetarian diets (63). Taking into account that an extreme dietary position such as a vegan diet or a vegan who consumed only uncooked and unprocessed plant-based food might show lacking micronutrient concentration, for example, calcium. Vegans require calcium-fortified foods that in combination and variety may help meet their daily calcium needs (64). However, in adults, as well as in children, it is not clear enough the mechanism behind bone health and dietary intake; therefore, further studies are needed to clarify this complex mechanism.

## The Influence of Physical Activity on Bone Health

Regular physical activity during growth seems to be one of the most important factors influencing peak bone mass. According to the International Osteoporosis Foundation (IOF), about 22% of men and 46% of women aged 50 years will experience osteoporotic fractures during the remainder of their lives (65). Thus, what should be done to optimize peak bone mass is to maximize the increase in BMD during the first 25 years by appropriate physical activity and to minimize the decrease in BMD after 40 years due to endocrine changes related to aging by regular physical activity. This easy strategy would reduce the occurrence in fractures later in life. However, so far, an exercise program for children and adolescents that will optimize peak bone mass in detail has not yet been defined.

The WHO has suggested that physical activity confers benefits for bone health in children and adolescents (66). Evidence comes from randomized small trials, which have some limits like confounding factors inherent to the cross-sectional studies (e.g., type of the exercise training, duration of the intervention, and group of intervention), which makes it difficult to figure out the osteogenic effects of physical activities. There are many systematic reviews that focus on bone strength in children and adolescents of both sexes in relation to physical activity (67). Some of them reported changes in bone structure rather than bone mass linked to enhanced bone strength.

In general, moderate-to-vigorous physical activity was linked to positive bone outcome especially in males, although discrepancy in the methodology assessment made it difficult to establish the amount and type of physical activity that might lead to favorable bone outcomes and therefore might exert the osteogenic action (68). What is recognized to exert an osteogenic action are interventions that must include high-intensity exercise of enough ground reaction forces (GRFs), to significantly increase bone mineralization and prevent osteoporosis and fragility fracture later in life. In fact, it was shown that high-impact jumping activities with GRFs from at least 3.5 and 8.8 × BW (10 min for two to three times per week) are effective in increasing BMD and/or BMC in children and adolescents, indicating that the most important factor is the intensity and not the duration of the stimulus (69). Usually, the intensity of an osteogenic exercise was expressed as GRFs, the combination of magnitude of force and speed by which it is applied (69, 70). It was demonstrated that activities with the most osteogenic potential have GRFs greater than 3.5 times BW (per leg) with peak force occurring in less than 0.1 s (71). Worthy of note is also a recent article about a project that assessed whether early elementary school children participating in 20 min of vigorous activity 3 to 5 days per week with 5 min of jumping component (GRFs between four and seven times’ body weight) would improve bone quality and muscular strength (70). The study confirmed the effectiveness of the program. Thus, a higher intensity level of physical activity achieves positive effects on BMC, BMD, and accretion (72). The importance of vigorous exercises as a favorable predictor of bone strength was further confirmed. In particular, 1 h per day of moderate-to-vigorous physical activity was able to improve bone strength in 6-year-old subjects (73). Also, participation in unstructured weight-bearing physical activity had strong and consistent positive effects on bone development (71, 74), further confirming that the high level of physical activity intensity is associated with higher modification in bone parameters during childhood (75, 76).

An evaluation study conducted from middle childhood to middle adolescence in more than 300 boys and girls in a 10-year longitudinal study confirmed that high participation in moderate-to-vigorous physical activity during childhood led to bone strength benefits in late puberty and that to improve bone BMC is necessary for vigorous physical activity interventions (77). Thus, vigorous physical activity was associated with BMC at skeletal sites from childhood to adolescence, and the effect was not modified by maturity or age (77). In fact, both engaging and maintaining high levels of vigorous physical activity participation during puberty in boys are associated with greater gains in bone mass and density (78). This is because at the age of 11–13 years, the growing bones of adolescents are more sensitive to mechanical loading than adult bones. Regarding the time spent on physical activity to achieve an osteogenic response, it was suggested that in adolescents, about 30 min per day of vigorous physical activity has shown benefits for the femoral neck BMD (79, 80), while 3 h per week could be enough to elicit an increase in bone mass (81). In fact, bone development is influenced by volume and intensity. Male adolescents (11–13 years) who participated in vigorous physical activity were positively correlated with increased whole body and lumbar spine BMC than sedentary, light, and moderate physical activity. Furthermore, the best benefits on BMC appear when 15 consecutive minutes of vigorous activity (like running) with respect to those who did it from 5 to 15 min and those who did it at less than 5 min. Therefore, with the same intensity of stimulus (vigorous activity), those who have carried out a longer duration showed better BMD than the rest of the subjects (82). However, it is important to note that not all activities have the same osteogenic effect on bone mass in children or adolescents. Such as in boys, long-term soccer participation, starting at a prepubertal age (Tanner stage 1–2), results in greater acquisition of bone mass and a lower accumulation of body fat (83). Bone acquisition is higher also in adolescent male footballers compared with swimmers and cyclists (84, 85). This suggests that weight‐bearing activities should be incorporated into training of swimmers in order to develop a stronger skeleton during adolescence. This would allow to optimize peak bone mass and reduce low bone status later in life. In addition, GRFs during impact exercises, especially during pubertal growth, play an important role in maximizing bone mineral gain (71). Among these activities, gymnastics has been shown to be particularly osteogenic for bone development in children because it is a high-impact activity and involves the subject at an early age during growth (86). However, intense athletic activity in growing and maturing gymnasts is often associated with inadequate dietary intake, which leads to relatively low fat mass (87) and consequently possible alteration of endocrine function (80). However, two major systemic reviews showed that prepubertal gymnasts have higher BMD and BMC than age-matched untrained controls. Therefore, gymnastics activities seem to be the one of most effective exercises for improving bone mineral gain in growing and maturing children (88, 89). Thus, the positive effect on bone accumulation seems to outweigh the possible negative influence of other characteristics.

What is important is to underline that optimal timing of a physical activity intervention differs depending on the stage of maturity of the pediatric population. In fact, BMC increases linearly, with no sex differences until the onset of the pubertal growth spurt (90, 91). Therefore, there are no differences in BMC and BMD between 6-year-old boys and girls (4) or younger subjects (3–5 years old) (5). However, some difference can be appreciated from the puberty phase onward where at the tibial diaphysis the cortical bone increases in size more for boys than for girls (92); therefore, the BMD varies considerably for the same chronological age among boys and girls. The peak of growth rate occurred earlier but is smaller in females than in males, and the female growth curve flattens before and with lower peak than men (93). Thus, males have a longer prepubertal period of growth because their pubertal growth spurt occurs 1 or 2 years later than in girls (94–96). However, this can cause a transient period of increased porosity at the cortex during periods of rapid growth. Particularly, boys demonstrate greater porosity than girls in this period (97). Bone thickness remains relatively stable until late puberty, as endosteal apposition is unable to keep pace with the rapid periosteal resorption that dominates the process of metaphyseal investing during periods of rapid longitudinal growth (98). The lag of bone thickness growth may contribute to increased bone fragility, and it could be a direct result of increased calcium demands, resulting in higher rates of intracortical bone turnover and increased porosity due to incomplete consolidation of bone (99).

Looking inside the sexual difference in BMC during adulthood, Baxter-Jones and collaborators analyzed 30 years later, in a prospective study, 82 female and 72 male children (age range 8 to 15 years) both more and less physically active (100). Participants returned for follow-up at age 23 to 30 years (2002–2006), and the cohorts were divided into active, average, and inactive groups. When compared with the inactive group, active females and males had greater adjusted BMC. In young adulthood, the male and female adolescent active groups were still more active than their peers and had still greater adjusted BMC. It was concluded that the skeletal benefits of physical activity in adolescents are maintained into young adulthood.

In conclusion, physical activity with certain characteristics seems to be better than others to improve bone mass. It should be dynamic (101) and vigorous (73, 75–78) with impact and load (71) to have a strong and consistent positive effect on bone development. However, further research to better clarify the modulatory role of the different activities on bone health in children and adolescents is necessary to improve and maintain bone health.

## Potential Explanatory Mechanism: PTH and Gut Peptides

**Figure 1:**
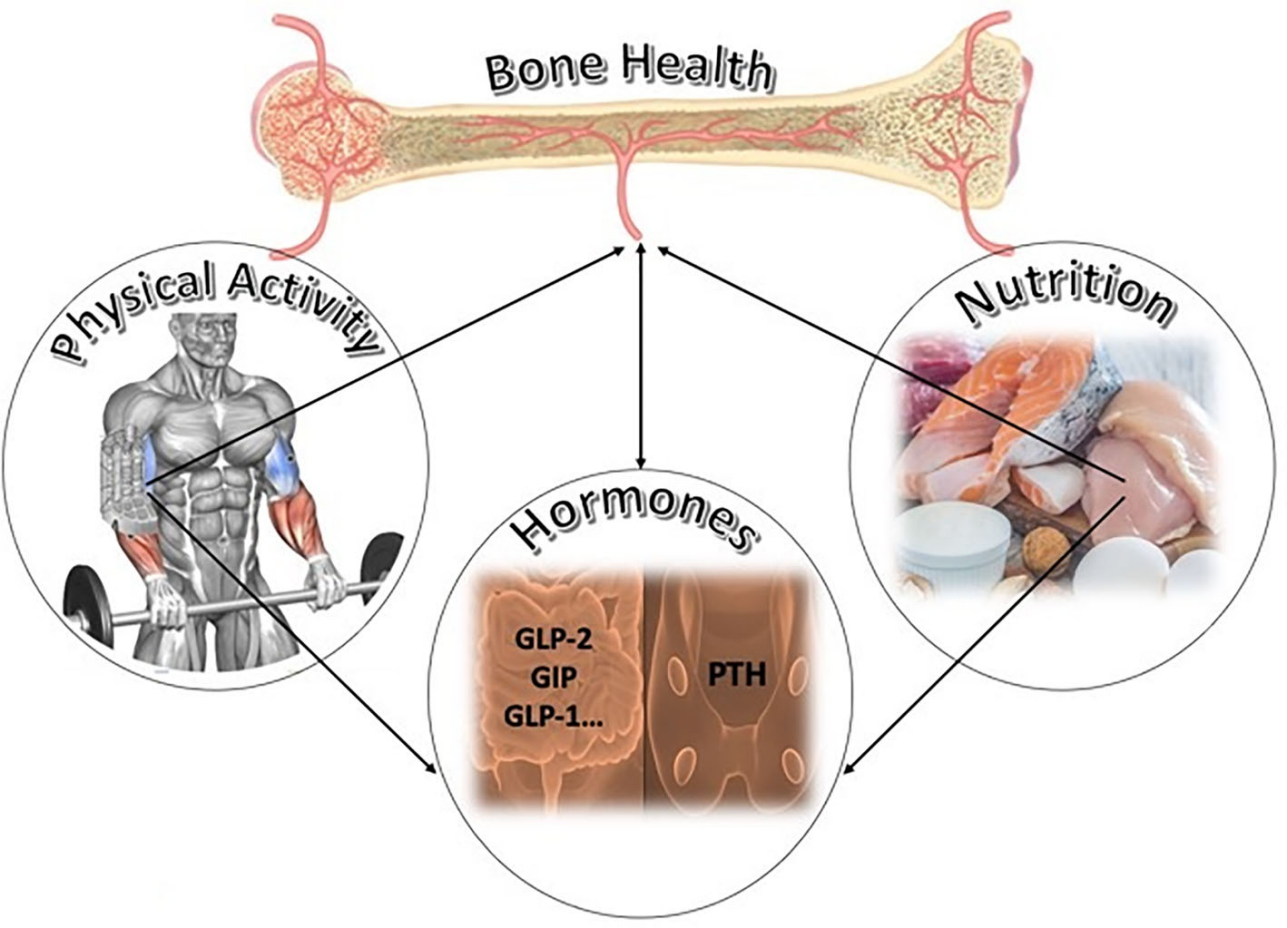
Potential key factors of bone homeostasis and remodeling: the bone is modulated by nutrition and by physical activity. Nutrition influences the release of hormones like gut peptides (GLP-1, GLP-2, and GIP) and modulates the secretion of parathyroid hormone (PTH). Both in turn influence bone remodeling. Physical activity, adequate in volume and intensity, could impact bone homeostasis by influencing hormones released by peripheral organs. This figure is our own creation.

Bone metabolism is influenced not only by hormones, such as the PTH, which is essential for the maintenance of calcium homeostasis, but also by hormones produced by other peripheral districts like the gastrointestinal tract (**Figure 1**). What is known about the influences of these factors on the bone and how are these affected by nutrients and physical activity?

The PTH has multiple effects on the skeleton. PTH stimulates osteocytes, osteoblasts, and their precursors. The physiological function of PTH is to maintain extracellular fluid calcium concentration and to prevent hypocalcemia. Therefore, with a feedback mechanism, PTH production is closely regulated by serum calcium concentration: PTH secretion increases whenever calcium concentration falls below normal, and the hormone is accountable for difference in blood calcium concentration, directly influencing the bone rather than kidney metabolism.

The hormone can prevent hypocalcemia at the cost of progressive bone destruction and loss of bone mineral (102).

Despite the role of PTH on bone homeostasis and remodeling, there are few studies about changes in PTH secretion and actions in healthy young subjects. These studies are not recent and did not focus on the effects of macronutrients and physical activity on PTH secretion. One of these studies shows the opposite variations of PTH and of 25-hydroxyvitamin D in a group of 42 children living in South Argentina (103). The association between a high level of PTH, a low level of vitamin D, and reduced bone mass was confirmed in a letter study conducted in pubertal and prepubertal Finnish girls (104). The secretion of PTH is modulated by vitamin D (105), suggesting that micronutrients could affect PTH secretion. Thus, also macronutrients could modulate PTH secretion in adolescence. For example, lower PTH concentrations and beneficial effects on bone size were observed in early pubertal children who have high fruit and vegetable intakes (106). This strongly suggests that nutrient supplementation influences PTH release in children and consequently bone homeostasis.

Physical exercise influences PTH release and increases PTH production, suggesting a possible key role in bone formation and adaptation to its mechanical features (107). However, research into the effects of exercise on PTH expression and secretion is still limited, and the study was conducted in adults. However, what was observed is that the increase in systemic PTH levels seems to depend on the type, intensity, and duration of exercise (108, 109). It was demonstrated that bone adaptation during exercise is not only a function of the dynamic loading but also PTH release and that PTH signaling contributes differently at structural and tissue levels (101). During exercise, there is an increase in calcium demand by the active muscle, but this increase is at the expense of bone mineralization at the periosteum (110). In fact, calcium supplementation during exercise may reduce bone resorption markers in adult female cyclists (58) and in general lessen the increase in PTH (57). In postmenopausal women, the time of calcium supplementation also seems to be important on the PTH release (111). In fact, the excess of calcium during exercise may impair systemic PTH release with a feedback mechanism (58). Further research is necessary to determine the effects of macronutrients and exercise alone and together on PTH secretion in children and adolescents to determine how this influences bone homeostasis and skeletal adaptations during growth. The gastrointestinal hormones have recently been seen to influence bone metabolism. The relationship between hormones secreted by the gut and bone is a recent study and opens the way to new interesting fields of research.

The bone and gut are strictly connected, and gut hormones respond to food intake, triggering bone resorption. Bone resorption is increased during the night compared with day, and diurnal suppression is eliminated by fasting, confirming the role of gastrointestinal hormones in controlling bone homeostasis (112)

Among the gut hormones, GIP, GLP-1, and GLP-2 are known to be involved in the regulation of bone turnover. These hormones have been extensively studied for their effects on glucose and lipid metabolism (113–118) and are of interest in the light of the interplay between the bone and glucose metabolism, which seems to be compromised in diabetes (119). The exact mechanism of action of these peptides on the bone is unclear. The GLP-1 and GLP-2 receptors are expressed in the immature human osteoblast cell lines MG-63 and TE-85 (120), but GLP-2 has not been identified in human osteoclasts or in any other bone-related cell types despite the impact of GLP-2 on osteoclast activity. GLP-2 is co-secreted with GLP-1 by L cells in the small and large intestine and was initially studied for its ability to stimulate mucosal growth and nutrient absorption in the intestine (121). GLP-2 treatment is associated with the reduction of serum and urinary markers of bone resorption in postmenopausal women, while bone formation appears not to be affected (122, 123). The GIP receptor is expressed in both osteoblast- and osteoclast-derived cell lines and increases the expression of type 1 collagen. It maintains osteoblast homeostasis with an anabolic effect on the bone. It also has an inhibitory effect on the bone resorption activity of PTH (124).

This information suggests that the gut hormones could impact bone health and affect bone quality. Thus, more studies are required to investigate the effects of diet and physical activity on secretion of gut peptides in children and the modulatory role, if any, to assure an optimal skeleton formation.

## Conclusion

There are several studies that have investigated in adulthood the best strategies in order to maintain good lean mass and bone mass by modification of diet and physical activity (125–130). Most of the adult studies have focused on osteoporosis risk subjects, postmenopausal women, and have analyzed the effect of minerals (mainly calcium), vitamin supplementation (mainly vitamin D), and nutrients, especially protein supplementation (131). There are few studies in childhood, and there are many questions to be answered. In fact, the skeleton is not an inert structure playing a supporting role for muscles and a protective role for inner organs like the brain. It is able not only to regulate its own physiology but also to influence energy metabolism by continuum interplay with the peripheral organs like the adipose tissue-derived hormones, the gastrointestinal tract-derived peptides, and insulin and by also producing the bone-derived peptides like osteocalcin and lipocalin-2. Nutrition and physical activity may influence the release of these factors in children. How this could impact bone health in children and adolescents is not completely known. Thus, studies that will address questions about the complex interplay between the bone, gut, white and brown adipose tissue, nutrition, and physical activity are required to provide in the near future new insight into this fascinating topic of metabolic endocrinology.

## Author Contributions

Conceptualization and writing: AA and SB. Original draft preparation: AA and SB. Review and editing: PP, SV, SB, AA, PD, and DK. All authors contributed to the article and approved the submitted version.

## Conflict of Interest

The authors declare that the research was conducted in the absence of any commercial or financial relationships that could be construed as a potential conflict of interest.

## Publisher’s Note

All claims expressed in this article are solely those of the authors and do not necessarily represent those of their affiliated organizations, or those of the publisher, the editors and the reviewers. Any product that may be evaluated in this article, or claim that may be made by its manufacturer, is not guaranteed or endorsed by the publisher.
